# Impact of different cell penetrating peptides on the efficacy of antisense therapeutics for targeting intracellular pathogens

**DOI:** 10.1038/srep20832

**Published:** 2016-02-10

**Authors:** Mostafa F. N. Abushahba, Haroon Mohammad, Shankar Thangamani, Asmaa A. A. Hussein, Mohamed N. Seleem

**Affiliations:** 1 Department of Comparative Pathobiology, College of Veterinary Medicine. Purdue University, West Lafayette, Indiana, USA, 47907; 2 Department of Animal Hygiene and Zoonoses, Faculty of Veterinary Medicine, Assiut University, Assiut, Egypt

## Abstract

There is a pressing need for novel and innovative therapeutic strategies to address infections caused by intracellular pathogens. Peptide nucleic acids (PNAs) present a novel method to target intracellular pathogens due to their unique mechanism of action and their ability to be conjugated to cell penetrating peptides (CPP) to overcome challenging delivery barriers. In this study, we targeted the RNA polymerase α subunit (*rpo*A) using a PNA that was covalently conjugated to five different CPPs. Changing the conjugated CPP resulted in a pronounced improvement in the antibacterial activity observed against *Listeria monocytogenes in vitro*, in cell culture, and in a *Caenorhabditis elegans* (*C. elegans*) infection model. Additionally, a time-kill assay revealed three conjugated CPPs rapidly kill *Listeria* within 20 minutes without disrupting the bacterial cell membrane. Moreover, *rpo*A gene silencing resulted in suppression of its message as well as reduced expression of other critical virulence genes (Listeriolysin O, and two phospholipases *plc*A and *plc*B) in a concentration-dependent manner. Furthermore, PNA-inhibition of bacterial protein synthesis was selective and did not adversely affect mitochondrial protein synthesis. This study provides a foundation for improving and developing PNAs conjugated to CPPs to better target intracellular pathogens.

Bacterial infections caused by multidrug-resistant pathogens are a daunting public health challenge that requires serious attention. For almost 80 years, antibiotics and their synthetic analogues have been the gold standard for treatment of these infections. However, the diminishing utility of current antibiotics in the face of rising bacterial resistance and the stagnant development of new antibiotics further underscores the urgent need for the development of alternative therapeutic options^1^. This scourge is further compounded by intracellular bacterial pathogens, such as *Mycobacterium*, *Salmonella, Listeria,* and *Brucella* that reside and thrive inside mammalian cells^2^,^3^,^4^. Treatment of infections caused by these intracellular pathogens is very challenging because most antibiotics are unable to access intracellular replicative niches and achieve the optimum therapeutic concentrations within the infected cells^3^,^4^,^5^. These challenges have sparked efforts to target intracellular bacteria utilizing different approaches^2^,^3^,^4^,^5^,^6^,^7^,^8^. One potential novel alternative therapeutic approach to treat infections caused by intracellular pathogens that has shown promise in recent years is silencing essential genes with a peptide nucleic acid (PNA)^6^,^7^,^8^,^9^. In addition to the hybridization affinity to their target DNA and RNA sequence, and specificity of PNA molecules to silence genes, these molecules are characterized by chemical and enzymatic stability conferred by their pseudopeptide backbone as well as low toxicity to host tissues^10^. However one significant limitation of these hydrophilic macromolecules is that their cellular uptake is controlled by the high selectivity imposed by cellular membranes^11^,^12^; this constitutes a major challenge for the successful utilization of PNA gene inhibition therapeutics to target intracellular pathogens^13^. Additionally, effective delivery of such large molecules across stringent bacterial cell walls can be a daunting task. Therefore, there is a need for an appropriate carrier system to deliver PNAs specifically to intracellular replicative niches and eliminate resident pathogen(s) effectively. Cell-penetrating peptides (CPPs), consisting of positively charged residues, have emerged as extremely efficient and crucial allies to PNAs and a wide range of cell-membrane impermeable cargos. These agents help molecules, such as PNAs, overcome challenging delivery barriers to permit their entry into infected cells^14^.

In this study, we investigated the effect of five different CPPs conjugated to PNAs that target the essential RNA polymerase α-subunit (encoded by *rpo*A gene) in the intracellular pathogen *Listeria monocytogenes.* We investigated the antibacterial activity of the PNAs, as well as their spectrum of activity against various clinical isolates of *Listeria* *in vitro*, in infected cell culture and in a *Listeria*-infected *Caenorhabditis elegans* model. We also studied the effect of silencing the *rpo*A gene and its impact on expression of Listeria virulence genes and evaluated the effect of PNAs on mitochondrial biogenesis. This study provides valuable insights into potential therapeutic applications of PNAs (conjugated to CPPs) for use as antimicrobial agents for the treatment of intracellular infections.

## Results

### Amplification and sequencing of rpoA 5′ terminal region

Bacterial strains included in this study are described in Table S1. We confirmed, via genomic sequencing, that the *rpo*A 5′ terminal region including the ribosomal binding site and the start ATG codon is conserved among *Listeria* clinical isolates (Tables S2 and S3). Using a complementary antisense PNA to the identified region within the *rpo*A gene, five different cell penetrating peptides were conjugated to the PNA (Table 1). These antisense constructs were subsequently tested *in vitro* and *in vivo* to confirm they were capable of inhibiting the *rpo*A gene and eradicate an infection caused by the intracellular pathogen *L. monocytogenes*.

### Minimum inhibitory concentration (MIC)

We explored the MICs of the five antisense constructs against clinical isolates of *L. monocytogenes* as presented in Table 2. All designed PNAs demonstrated bactericidal activity against *Listeria* except PKFF which worked as bacteriostatic. As shown in Table 2 the most effective PNA was PRXR followed by PTAT and PRFR. The MIC_50_ of PRXR was found to be 1 μM and the MIC_50_ of PTAT and PRFR was found to be 2 μM and 4 μM, respectively. PANT and PKFF were less effective inhibiting bacterial growth with MIC_50_ of 32 μM. Neither the free PNA nor the free CPPs possessed antimicrobial activity up to 32 μM. KFF and ANT CPP showed antimicrobial activity with MIC_50_ of 64 μM.

### Bacterial reduction

After confirming the MIC for each PNA construct, concentration-dependent bacterial reduction was determined against *L. monocytogenes* F4244. Table S4 presents the average number of viable bacteria present after treatment and the corresponding log reduction associated with each PNA (relative to the control). As expected, PRXR (1 μM) produced the greatest reduction (Log_10_ 2.51) in bacterial count followed by PTAT with bacterial reduction of (Log_10_ 1.1) at the same concentration. Both PNAs cleared *Listeria* (9.39 log_10_ reduction) at 4 μM. PRFR showed significant reduction of (log_10_ 1.91) in bacterial count at 2 μM and bacterial clearance at 8 μM. PANT showed significant reduction of (log_10_ 1.3) in bacterial count at 8 μM and bacterial clearance at 32 μM, respectively. PKFF was the least effective, as predicted from the MIC results, and only produced a reduction of (log_10_ 0.98) and (log_10_ 2.78) in bacterial count at 16 μM and 32 μM, respectively.

### Time kill kinetics

**Figure 1:**
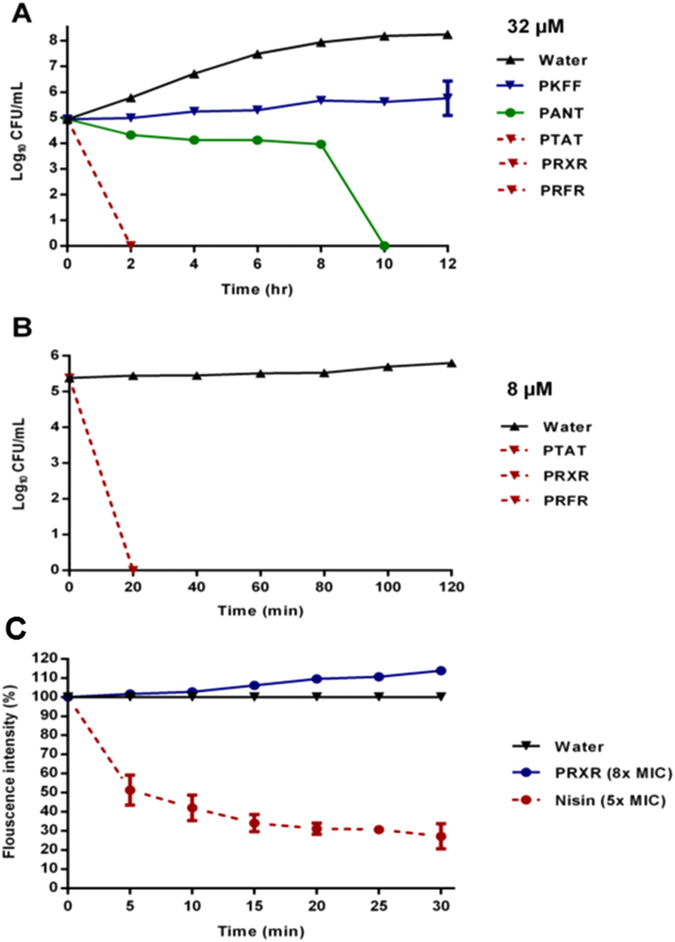
Time-kill analysis of PNAs against *L. monocytogenes* F4244. (**A**) 32 μM of the five PNAs at 37 °C were incubated for 12 hours and samples were collected every two hours. (**B**) 8 μM of PTAT, PRXR, and PRFR at 37 °C were incubated for 2 hours and samples were collected every 20 minutes. The results are presented as means ± SD from two independent experiments (n = 3). (**C**) Permeabilization of the cytoplasmic membrane of *L. monocytogenes* F4244 indicated by percent of calcein leakage for 30 min exposure to PRXR and nisin. Samples were collected every 5 minutes. The results are given as means ± SD (n = 3; data without error bars indicate that the SD is too small to be seen).

After confirming the antisense constructs possessed excellent antimicrobial activity against *L. monocytogenes*, we next assessed the killing kinetics of the PNAs. We exposed *L. monocytogenes* strain F4244 to 32 μM PNAs and control (water) for 12 hours. Samples were taken every 2 hours. As depicted in Fig. 1A, PRXR, PTAT and PRFR showed fast bactericidal activity with complete eradication of *Listeria* by 2 hours. PANT showed slow bactericidal activity with complete clearance after 10 hours. PKFF showed bacteriostatic effect with no reduction in bacteria after 12 hours incubation comparing to starting inoculum. As the initial time-kill assay revealed complete eradication of bacteria within two hours for PTAT, PRXR, and PRFR, a second time kill assay was performed with a lower concentration of 8 μM to determine how rapidly these constructs kill bacteria. Samples were taken every 20 minutes. Interestingly, we found that these three PNAs were capable of completely eliminating a high starting inoculum of bacteria (2.4 × 10^5^ CFU/ml) within 20 minutes at 8 μM concentration (Fig. 1B).

### PNA does not target the integrity of the bacterial cell membrane

We suspected that the rapid bactericidal activity of the PRXR, PTAT and PRFR PNAs was due to disruption of the bacterial cell membrane. To examine this, a calcein leakage assay was performed to investigate the effect of PNA on the integrity of the *Listeria* cell membrane. Cell membrane disruption leads to leakage of calcein from damaged preloaded bacterial cells, resulting in a reduction in the fluorescence intensity observed in both a concentration- and time-dependent manner^15^. Nisin, a known membrane-disrupting agent, was used as a positive control. At 8 × MIC, the PRXR did not result in leakage of calcein from bacterial cells (matching the result obtained for the untreated control); in contrast, the antimicrobial peptide nisin, at 5 × MIC, led to more than 72% leakage within 30 minutes (Fig. 1C). This result indicates the antisense construct does not target the integrity of the bacterial cell membrane.

### PNA inhibits rpoA gene expression and expression of virulence genes

**Figure 2:**
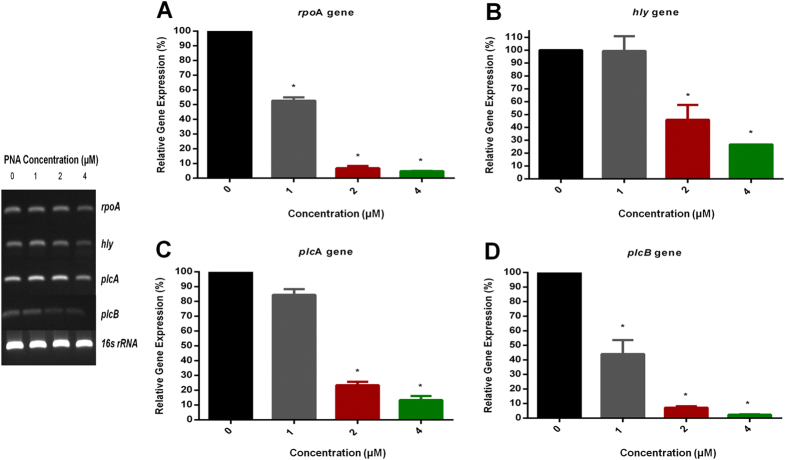
Concentration-dependent reduction of *rpo*A, *hl*y, *plc*A and *plc*B expression after treatment with PTAT. Bacterial cultures were treated with 1, 2 and 4 μM PTAT for 3.5 hours at 37 °C. Total RNA was extracted from the treated and untreated cultures. The levels of mRNA were determined by RT-PCR. (**A**) The level of *rpo*A expression. (**B**) The level of *hl*y expression. (**C**) The level of *plc*A expression and (**D**) the level of *plc*B expression. *P* value of (^*^P ≤ 0.05) is considered as significant.

Real-Time qRT-PCR was utilized to determine the effect of PTAT on *rpo*A expression and the subsequent suppression of three important genes, *hly*, *plc*A and *plc*B encoding for *Listeria* virulence factors (Table S5). PTAT significantly inhibited the expression of *rpo*A in a concentration-dependent manner; at the lowest tested concentration of 1 μM, a 50% reduction in *rpo*A gene expression is observed (Fig. 2A). Interestingly, repression of *rpo*A led to significant repression of important virulence genes such as *hly*, *plcA, and plcB* (Fig. 2B–D). When tested at 2 μM PTAT, the *hly* gene was downregulated by more than 50% while both *plc*A and *plc*B gene expression decreased by more than 70%. These results indicate that down regulation of the *rpo*A gene in *L. monocytogenes* (with the peptide nucleic acid) has a suppressive effect on other essential genes and virulence factors.

### PNA does not inhibit Mitobiogenesis

**Figure 3:**
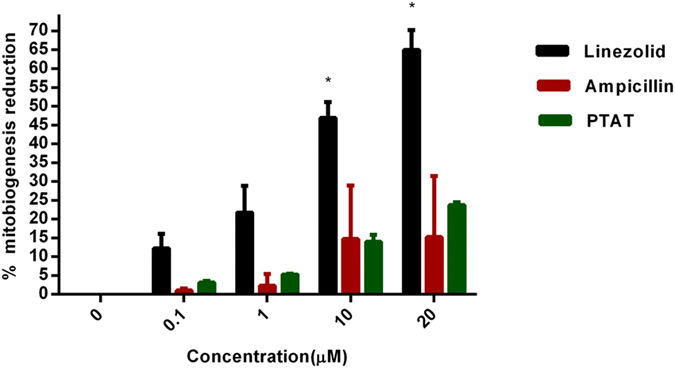
Effect of PNA, linezolid and ampicillin on mitobiogenesis. In cell- ELISA was carried out in the presence and absence of these drugs, and the levels of mitochondrial (mt)-DNA encoded protein (COX-I) and nuclear-DNA encoded protein (SDH-A) in J774A.1 were quantified. Ratio of COX-I and SDH-A was calculated and the results were shown as percent inhibition of mitochondrial biogenesis.

Antimicrobials that target microbial protein synthesis are considered excellent choices for the treatment of virulence and toxin-mediated bacterial infections^16^,^17^,^18^,^19^. In addition to the suppression of toxins and virulence factors, these antimicrobials also reduce excessive host-inflammatory responses associated with these toxins^20^,^21^. Hence, protein synthesis inhibitors are often preferred in clinical practice for the treatment of toxin-associated bacterial infections^16^,^17^,^18^,^19^. However, due to concern about possible toxicity to eukaryotic mitochondria, as observed with many antibacterial protein synthesis inhibitors such as linezolid and chloramphenicol^22^,^23^, we tested the effect of PNA on mitochondrial protein synthesis directly within mammalian cells. In-cell ELISA was performed in J774A.1 cells treated with PNA, ampicillin and linezolid for three days to detect the levels of mtDNA-encoded COX-I and nDNA-encoded SDH-A proteins. Results depicted in Fig. 3 indicate that the PNA behaves similar to the negative control antibiotic ampicillin which does not significantly inhibit mitobiogenesis; this confirms that the PNA does not interfere with the mitochondrial protein synthesis process. Linezolid, in contrast, inhibited mitochondrial protein synthesis by 65%. These results provide valuable information about the PNA’s safety profile against mammalian cells and the lack of interference with mitobiogenesis.

### Antimicrobial activity of PNA in infected cell culture

Due to the fact that *Listeria* infects, resides and replicates inside host cells, it was important to test the activity of our PNAs against intracellular *Listeria*. As presented in Table 3, PRXR displayed the most potent activity with significant reduction (Log_10_ 1.78) in intracellular *Listeria* at 2 μM and complete clearance of intracellular *Listeria* at 8 μM. PTAT and PRFR at 2 μM demonstrated significant reductions of (Log_10_ 0.78) and (Log_10_ 0.84), respectively. PANT and PKFF were the least effective in eradicating intracellular *Listeria* producing (Log_10_ 2.07) and (Log_10_ 0.6) reductions, respectively, at 8 μM.

### Activity of PNA constructs *in vivo* using *C. elegans*

**Figure 4:**
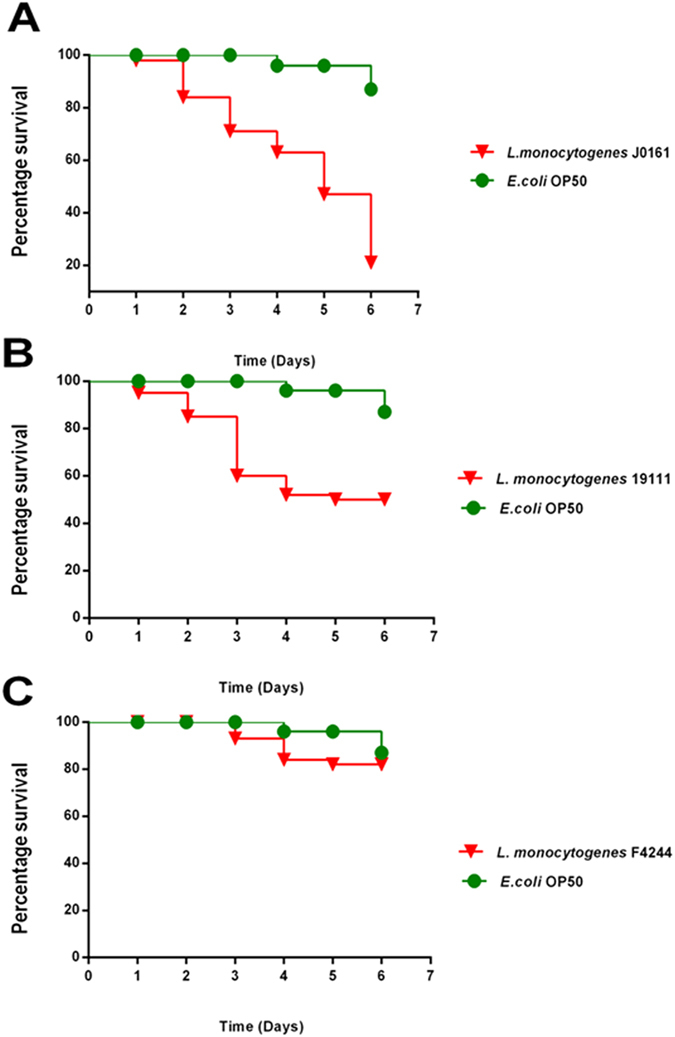
Killing of *C. elegans* by different strains of *L. monocytogenes*. Worms were grown on lawns of bacteria for 8 hours before washing and transferring to microtitre plate. (**A**) Worms fed *E. coli* OP50 or *L. monocytogenes* J016. (**B**) Worms fed E. coli OP50 or L. monocytogenes 19111. (**C**) Worms fed E. coli OP50 or *L. monocytogenes* F4244.

**Figure 5:**
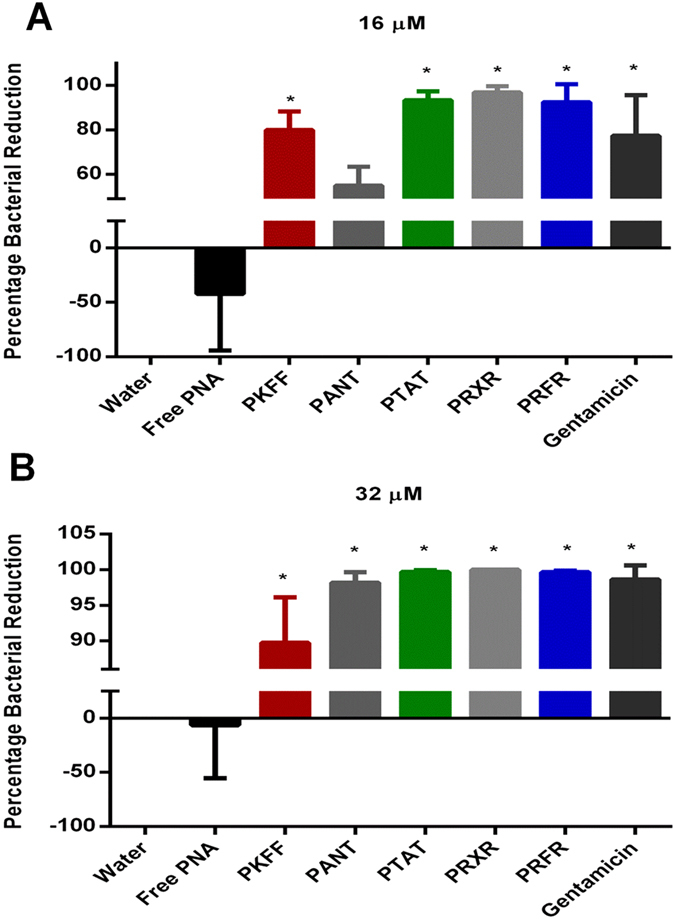
Evaluation of antimicrobial efficacy of PNAs in *C. elegans* model at 16 μM (**A**) and 32 μM (**B**). *L. monocytogenes* J0161 infected L4-stage worms were treated with PNAs and antibiotic for 18 h. Worms were lysed and the CFUs were counted and the percent bacterial reduction per worm in treated groups were calculated in relative to the untreated control groups. 10 worms in triplicates were used for each treatment. The results are presented as means ± SD from two independent experiments. Data without error bars indicate that the SD is too small to be seen. *P* values of (*≤ 0.05) are considered as significant.

Confirmation of PRXR, PTAT, and PRFR’s ability to inhibit *Listeria* growth *in vitro* and in cell culture led us to assess the PNAs’ ability to treat an infection caused by *L. monocytogenes in vivo*. To test the efficacy of PNA *in vivo* we used *Listeria*-infected temperature-sensitive sterile mutant strain *C. elegans* AU37 [sek-1(km4); glp-4(bn2) I]. This strain is sterile at room temperature and can lay eggs only at 15 °C. Furthermore, mutation in the sek-1 gene of the p38 mitogen-activated protein kinase pathway (p38 MAPK pathway), makes this particular *C. elegans* strain more susceptible to *Listeria* infection. Initially, the pathogenicity of three strains of *Listeria* (F4244, J0161 and ATCC19111) was tested in *C. elegans* as described before^24^ to determine the most virulent strain to use for this study. Figure 4 shows the pathogenicity of three strains of *Listeria* (F4244, J0161 and ATCC19111) in *C. elegans.* Strain J0161 (clinical isolate from Human listeriosis) was the most pathogenic strain, killing 80% of worms in 6 days. This strain was selected for testing PNA treatment efficacy *in vivo*. Each antisense construct was tested at two concentrations (16 μM and 32 μM) to treat *C. elegans* infected with *L. monocytogenes* J0161 for 18 hours. PTAT led to a significant reduction in bacteria both at 16 μM and 32 μM correlating to a 1.11 and 2.67 log CFU reduction, respectively (Fig. 5 and Table S6). PRFR resulted in 1.3 and 2.52 log CFU reduction at 16 μM and 32 μM, respectively. A 1.89 log CFU reduction was achieved upon application of PANT at 32 μM and PKFF caused a significant reduction both at 16 and 32 μM concentration with 0.71 and 1.03 log CFU reduction, respectively. The best performing antisense construct *in vivo* was PRXR. Complete bacterial clearance was achieved by applying PRXR at 32 μM; this same construct (at 16 μM) produced a 1.91 log reduction in *L. monocytogenes* in infected worms.

## Discussion

As an intracellular pathogen, *L. monocytogenes* manipulates the host cell machinery to gain entry into multiple cell types, escape phagosomes quickly, and multiply in the cytosol of infected host cells^25^,^26^. This permits the pathogen to escape detection and eradication by the host immune response making clearance of infection very challenging. Traditional antibiotics are often incapable of passively permeating host cells to kill intracellular pathogens, making it difficult to treat infected patients. This limitation combined with the increasing prevalence of resistance to conventional antibiotics has led researchers to search for alternative agents that can be used as novel treatments. One promising agent that has emerged recently are peptide nucleic acids given their unique and targeted mode of action in silencing expression of essential genes. Conjugating these PNAs with an effective cell penetrating peptide permits the entry of PNAs into cells in order to eradicate intracellular pathogens harboring inside these cells.

RNA polymerase (RNAP) is conserved among prokaryotes and plays a vital role as a key enzyme in gene expression and protein synthesis. Moreover, despite the similarities between prokaryotic and eukaryotic RNAPs in their pervasiveness, structure, and function, there is no extensive sequence homology between the both of them^9^,^27^,^28^. These reasons highlight why bacterial *rpo*A is an excellent target to silence using PNAs.

Previously our research group has investigated the capability of an anti-*rpo*A PNA and an anti-*rpo*D PNA to inhibit growth of *L. monocytogenes*^5^; this study revealed that the (KFF)_3_ K-anti-*rpo*A PNA (PKFF) is more potent than the (KFF)3K-anti-*rpo*D PNA. Utilizing this information, we engineered five antisense agents (targeting the same genetic sequence of the *L. monocytogenes rpo*A gene) conjugated to five different cell penetrating peptides (CPPs) - (KFF)_3_K, antennapedia, TAT, PRXR, and PRFR. The CPPs were selected based upon established studies. The synthetic CPP (KFF)_3_K has been used for delivery of PNAs in many Gram-positive bacteria including *L. monocytogenes* F4244^5^, methicillin-resistant *S. aureus*^27^,^29^ and *Streptococcus pyogenes*^30^. Antennapedia is a known efficient vehicle to deliver antisense molecules into mammalian cells^31^,^32^,^33^. TAT has been shown to enhance the antisense effect of anti-*gyr*A PNA against *Streptococcus pyogenes*^30^. Multiple studies have demonstrated that the (RXR)_4_XB CPP acts as a potent tool for intracellular delivery of DNA analogues into a broad range of Gram-positive and Gram-negative bacteria^28^,^34^,^35^. (RFR)_4_XB has been reported to be an effective carrier for intracellular delivery of antisense components into *E. coli*^34^. The mechanism of uptake for CPPs and their potentiality for intracellular delivery of cargo molecules are controversial^36^. Also, the nature of the cargo molecules affects the uptake properties of these peptides. Even with these challenges, CPPs are considered one of the few alternatives available to significantly enhance the effect of antisense agents in various applications^36^,^37^.

Keeping the above points in mind, the objective of the present study was to assess the impact of different CPPs conjugated to the same anti-*rpo*A PNA to target and eradicate intracellular pathogens both *in vitro* and *in vivo*. In general, PTAT, PRXR and PRFR were found to be more potent than either PKFF or PANT. When compared to PKFF, all the three PNAs exhibited a 16-32 fold improvement in the MIC against *L. monocytogenes*. In the present study, the free unconjugated PNA did not possess antimicrobial activity against *L. monocytogenes* which would reinforce the notion that poor cellular uptake of PNAs remains a major constraint for utilization of PNA therapeutics to effectively target intracellular pathogens. Utilization of appropriate CPPs does help to facilitate the cellular uptake of the designed PNA into *L. monocytogenes* as noted by the improved antibacterial activity of the anti-*rpo*A PNA-CPP constructs tested against *L. monocytogenes*. However, the CPPs themselves (TAT, RXR and RFR) showed no growth inhibition in pure culture against *L. monocytogenes* F4244. This confirms that the antibacterial action of the antisense constructs relies directly on the inclusion of an effective PNA that can silence the expression of the target gene(s).

After confirming the CPP-PNA constructs exhibited potent antimicrobial activity against *L. monocytogenes*, we next set out to determine if these constructs exhibit bacteriostatic or bactericidal activity. A time-kill assay was utilized and demonstrated that four constructs (PANT, PTAT, PRXR and PRFR) do in fact exhibit bactericidal activity (resulting in complete eradication of bacteria within 10 hours). Interestingly, the PTAT, PRXR and PRFR PNAs were capable of completely eliminating a high initial inoculum of *L. monocytogenes* within 20 minutes. The rapid bactericidal activity noted with these PNAs raised a question regarding whether these particular constructs were exhibiting a specific antisense effect or were potentially mimicking the effect of antimicrobial peptides as non-specific cell membrane disrupters^15^. To address this question, two experiments were performed (1) to determine the MIC of PRXR against MRSA USA300 in pure culture (to confirm its antibacterial activity was limited to *L. monocytogenes*) and (2) to conduct a calcein leakage assay (using 8 × MIC of PRXR) to determine if this construct was capable of disrupting the bacterial cell membrane. When tested against MRSA, PRXR was incapable of inhibiting bacterial growth even up to a concentration of 16 × MIC (data not shown), indicating that the construct has selective activity against the target pathogen (*L. monocytogenes*). The calcein leakage assay further confirmed that the PRXR does not permeabilize the bacterial cell membrane. Thus it appears from these results that the biological action of these CPP-PNA constructs is likely due to silencing the expression of the target gene (*rpo*A). This postulate was confirmed using Real-Time qRT-PCR to assess for the level of gene expression in the presence of increasing concentration of PNA.

Our Real-Time qRT-PCR analysis revealed that the CPP-anti-*rpo*A constructs were in fact successful in reducing the expression of *rpo*A, in a concentration-dependent manner. As the concentration of the PTAT increased (from 1 to 4 μM), the percentage of gene expression of *rpo*A diminished significantly (from more than 50% to less than 5%). This clearly proves that the *rpo*A gene is critical for sustaining the viability of *L. monocytogenes*. Furthermore, *rpo*A gene inhibition leads to a reduction in the expression of other major virulence genes required for *L. monocytogenes* pathogenesis. Listeriolysin O (LLO), encoded by the *hly* gene, is considered the main virulence factor of *L. monocytogenes* and is a cytolytic toxin that forms pores in vacuolar membranes causing a passive flux of ions and macromolecules. This leads to quick vacuolar lysis and bacterial release into the cytosol^38^,^39^,^40^. In addition to LLO, *L. monocytogenes* secretes two phospholipases, PI-plc (encoded by *plc*A) and PC-plc (encoded by *plc*B). They are well-known virulence factors that work in concert with LLO in order to help the bacteria to escape from vacuoles present inside host cells^40^,^41^,^42^,^43^,^44^,^45^,^46^. At a concentration of 1 μM, the PTAT construct was able to produce a 43.7% reduction of the *rpo*A message and subsequently a 55.9% reduction in *plc*B gene expression. As the concentration of PNA was increased, the degree of gene expression declined sharply for all tested genes.

As noted earlier, antimicrobials that target protein synthesis in bacteria are considered excellent choices for the treatment of virulence and toxins-mediated bacterial infections^16^,^17^,^18^,^19^ with one major caveat. These particular agents, including linezolid, often are toxic to mitochondria present in mammalian cells. Thus it was critical for us to assess whether our antisense constructs, given their impact on bacterial protein synthesis, had a negative impact on the mitochondria. The effect of PNAs on mitochondrial protein synthesis was detected by measuring the level of mtDNA-encoded protein COX- and nDNA-encoded protein SDH-A post-treatment. The PNA demonstrated no significant inhibition of mitobiogenesis, similar to the effect of ampicillin, which does not interfere with the mitochondrial protein synthesis process. These results provide valuable information that our antisense constructs do not interfere with mitobiogenesis.

After confirming the mechanism of action of the tested PNAs was suppression of *rpo*A gene expression, we moved next to confirm that the PNAs could retain their activity *in vivo*, both inside macrophages (where *L. monocytogenes* reside normally) and using *C. elegans* as a unique animal model for infection. Therapeutic agents can often possess potent activity *in vitro* but fail to demonstrate efficacy in cell culture models or in *in vivo* studies. Therefore, to assess if these CPP-PNA constructs could retain their activity *in vivo*, we first tested our antisense constructs against *L. monocytogenes* infected murine macrophage cells. A significant reduction of the intracellular bacteria was achieved by PTAT, PRXR and PRFR PNAs at all tested concentrations. The antibacterial effect of the PNAs was concentration-dependent with no microscopically observed cytotoxicity. Additionally, complete clearance of the infection was observed when using the PRXR construct. Interestingly, PANT is more potent against *L. monocytogenes* F4244 in infected cell culture as compared to results obtained in pure culture.

*In vitro* models cannot be used as a suitable substitute to simulate the actual environment present within an infected human host. Therefore, there is a need to identify alternative models to investigate the effect of antimicrobial compounds in a living system. In recent years, *C. elegans* has been established as a powerful model for *in vivo* screening of antimicrobials against multiple pathogens^47^,^48^,^49^,^50^,^51^. This provided the impetus for testing the effectiveness of our antisense constructs in *C. elegans* infected with *L. monocytogenes*. Our antisense constructs retained their potent antibacterial activity *in vivo,* significant reduction in bacteria (in relation to the untreated control) observed for PTAT, PRXR, PRFR and PKFF, when tested at 16 and 32 μM. This effect was concentration-dependent as a higher concentration of PRXR was needed to clear the infection. Significant reduction was achieved by application of PANT only at 32 μM concentration. Additionally, our study revealed that there is a significant difference between using free unconjugated PNA as compared to PNAs conjugated to a CPP for treatment of the infected *C. elegans*. This confirms the important role of CPPs for intracellular delivery of antisense constructs to reach the target microorganism and silence gene expression. Though complete eradication of bacteria in infected worms was not achieved (except by using a high concentration of PRXR), this may be attributed to several factors. Among these factors include the bioavailability of PNAs inside the worms, non-specific binding and bio-distribution of the PNAs, high molecular weight and polarity of the constructs (limiting their ability to cross membrane barriers), in addition to the system complexity of *C. elegans*. Collectively these factors may explain the reduced antisense effect observed with the PNAs in the *C. elegans* model when compared with the results obtained in pure culture and cell culture^8^,^52^.

In this study, we demonstrate that the bacterial *rpo*A gene, which encodes the α-subunit of RNA polymerase, is a critical gene for the viability of *L. monocytogenes*. Antisense targeting can successfully inhibit the expression of this gene and lead to direct bacterial cell death. This inhibition effect is concentration-dependent. Our investigation confirmed the antisense constructs were capable of killing *L .monocytogenes* in pure culture, in infected macrophage cells, and in a *C. elegans* animal model. The rapid bactericidal effect observed is due to silencing of *rpo*A gene expression and not due to bacterial membrane disruption. Based upon our *in vitro* and *in vivo* results, we confirmed that (RXR)_4_XB followed by TAT and (RFR)_4_XB, are considered the most suitable vehicles to conjugate with the anti-*rpo*A peptide nucleic acid to target cells infected with *L. monocytogenes*. This work lays the foundation for investigating these CPPs in conjunction with other PNAs to silence expression of essential genes in intracellular pathogens.

The present study validates the notion that the *rpo*A gene is an encouraging target for the development of the antisense therapeutics for effective targeting of intracellular pathogens like *Listeria*. Additionally, we confirmed that selecting an appropriate carrier/vehicle to deliver the PNA is very important in maximizing the antisense effect observed against the target microorganism.

## Materials and Methods

### Chemicals, reagents and kits used

Trypticase soy broth (TSB), trypticase soy agar (TSA), brain-heart infusion (BHI), Luria-Bertani (LB) broth and Luria-Bertani agar were purchased from BD/Difco (Sparks, Maryland, USA). Fetal bovine serum (FBS) and calcein AM were purchased from Life Technologies (Grand Island, NY, USA). Dulbecco’s modified Eagle’s medium (DMEM), Dulbecco’s Phosphate Buffered Saline (PBS), nisin, chloroform, isopropanol, agarose, ethidium bromide, Tris-Borate-EDTA buffer, free water and primers were purchased from Sigma-Aldrich Co. (St. Louis, MO, USA). Gentamicin, TRIzol Max Bacterial RNA Isolation Kit, SYBR Green PCR Master Mix, SuperScript II Reverse Transcriptase, 1 kb plus DNA ladder were purchased from Invitrogen (Carlsbad, CA, USA). TURBO DNA-free Kit and DEPC-treated water were purchased from Ambion (Foster city, CA, USA). Random hexamers (Applied Biosystems, Carlsbad, CA, USA), QIAquick Gel Extraction Kit (Germantown, MD, USA) and In-Cell ELISA Kit (MitoSciences Inc., Eugene, OR, USA) were also used in this study.

### Bacterial strains and C. elegans

Methods were carried out in accordance with the approved guidelines. Bacterial strains included in this study are described in Table S1. The temperature-sensitive sterile mutant strain *C. elegans* AU37 [sek-1(km4); glp-4(bn2) I] was used for testing the efficacy of PNAs in an *in vivo* model of *Listeria* infection. Worms were maintained on nematode growth media (NGM) plates seeded with *Escherichia coli* OP50. For infection, worms were maintained on LB agar plates seeded with *L. monocytogenes* J0161.

### Amplification and sequencing of rpoA 5′ terminal region

DNA was extracted from *Listeria* isolates by incubating ~10^9^ colony forming units (CFU) of *Listeria* in water at 95 °C for 10 minutes. The supernatant (containing DNA) was used for amplification of the *rpo*A 5′ terminal region including the ribosomal binding site and the start ATG codon by PCR. Primers RpoA-seqF and RpoA-seqR, indicated in Table S7, were used for amplification. Detection of PCR-amplified product was performed by electrophoresis on a 1% (wt/vol) agarose gel stained with ethidium bromide. Bands of DNA stained with ethidium bromide were visualized after exposure of the gel to ultraviolet (UV) light. Amplified PCR products were extracted from the gel using the QIAquick Gel Extraction Kit. The purified DNA fragments were sent for nucleotide sequencing at the Purdue Genomics Facility (ABI 3137XL low-throughput capillary machine) using the forward and reverse primers. Sequence alignment of the *rpo*A 5′ terminal region was performed with the BLAST alignment program present in the GenBank database (National Institutes of Health).

### Cell penetrating peptides and PNAs

Cell penetrating peptides ((KFF)_3_K^6^,^7^,^10^, antennapedia^31^,^33^, TAT^30^, (RXR)_4_XB^28^,^34^,^35^, and (RFR)_4_XB^34^, used in this study are presented in Table 1. Peptides were synthesized and purified by GenScript (Piscataway, NJ, USA). The 12 nucleotide target sequence of the PNA was chosen to be complementary to a specific target region of *rpo*A gene that exhibited previous success^5^. The CPPs (antennapedia, TAT, (RXR)_4_XB, and (RFR)_4_XB) were covalently conjugated with the PNA. The (KFF)_3_K CPP has been reported before^5^. PNAs were synthesized and purified by PNA Bio Inc. (Thousand Oaks, CA, USA). Free PNA without CPP was synthesized and used as a control.

### Minimum inhibitory concentrations (MICs) and bacterial log reduction determination

The MICs of conjugated PNAs, free PNA, CPPs and untreated control against *L. monocytogenes* were determined using the broth microdilution method according to the Clinical and Laboratory Standards Institute (CLSI) guidelines with the following modifications. Briefly, low binding clear microcentrifuge tubes (USA scientific, Inc. Ocala, FL) were used in triplicates for each reaction instead of traditional 96- well plates. The MIC was recorded as the lowest concentration where no turbidity was observed in the tubes. After a 16 hour incubation period (of test agent with bacteria), the number of viable bacteria was enumerated by serial dilution and counting on TSA plates. The MICs were repeated at least twice.

### Time kill assay

*L. monocytogenes* F4244 in logarithmic growth phase was diluted to ~10^5^ CFU/ml and incubated with 32 μM of the five PNAs (in triplicates) at 37 °C for 12 hours. Samples were collected every two hours, serially diluted, and plated onto TSA plates. Plates were then incubated wat 37 °C for 24 hours before viable CFUs were determined.

As the initial time-kill assay revealed complete eradication of bacteria within two hours for PTAT, PRXR, and PRFR, a second time-kill assay was performed for these three constructs using a lower concentration (8 μM). Samples were collected every 20 minutes and processed as above.

### Calcein leakage assay

To investigate the effect of PNA on the integrity of the *Listeria* cell membrane, the leakage of the preloaded fluorescent dye, calcein, was monitored and quantified as described before with a few modifications^15^. Briefly, 20 ml of logarithmic growth phase (OD_600_ = 1.0, ~10^9^ CFU/ml) *L. monocytogenes* F4244 was centrifuged and bacteria were resuspended in 9 ml of sterile PBS plus 1 ml of BHI. 3 μM of calcein AM dye was added to the bacterial suspension prior to covering the suspension with aluminum foil and incubating for one hour at 37 °C. After 10 minutes centrifugation at 3,000 × *g*, the supernatant was discarded. The calcein-loaded bacterial cells were resuspended in 30 ml of sterile PBS and 100 μL calcein-loaded bacteria were distributed in a 96-well microtiter plate (in triplicate). PRXR was added in a concentration equal to 8 × MIC (8 μM). Nisin 5 × MIC (10 μg/ml) and sterile water were used as positive and negative controls, respectively. Intensity of fluorescence (calcein leakage) was detected every 5 minutes for 30 minutes using a fluorescence plate reader (FLx800 model BioTek® Instruments, Inc. Winooski, Vermont) with the excitation and emission filters adjusted to 485 nm and 520 nm, respectively.

### Measuring gene expression in PNA-treated Listeria

To detect the expression of *rpo*A and assess the effect on expression of virulence genes including *hly*, *plc*A and *plc*B, a quantitative real-time PCR experiment was carried out (proposed function of these genes are presented in Table S1). Briefly, PTAT at a final concentration of 1, 2 and 4 μM was incubated with 10^9^ CFU/ml of *L. monocytogenes* F4244 for 3.5 hours at 37 °C. Total RNA was extracted from the treated and untreated cultures using the TRIzol Max Bacterial RNA Isolation Kit. The phase separation step, using chloroform, was repeated twice to minimize the carryover of phenol and guanidine isothiocyanate. To eliminate genomic DNA contamination, the RNA samples were subjected to Turbo DNase treatment (Ambion, Grand Island, NY, USA). The absence of genomic DNA was confirmed using conventional PCR and running samples on a 1% agarose gel. RNA quantity and purity was determined using the NanoDrop 1000 (Thermo Fisher Scientific, Waltham, MA, USA).

For first strand cDNA synthesis, seventy nanograms of Turbo DNase treated RNA were reverse transcribed using random hexamers (Applied Biosystems, CA, USA) and SuperScript II Reverse Transcriptase (Invitrogen, Carlsbad, CA, USA), according to the manufacturer’s protocol. Specific primers for the 16 s *rRNA, rpo*A*, hly*, *plc*A and *plc*B genes were designed and purchased commercially (Sigma-Aldrich, St. Louis, MO, USA) (Table S7).

Each cDNA reaction was conducted using duplicate samples. Amplification was performed in an ABI 7300 Real-Time PCR System (Applied Biosystems, CA, USA) using the following conditions; 95 °C for 10 minutes as an initial step for DNA polymerase activation, 95 °C for 15 seconds for melting and 60 °C for one minute for annealing/extension (40 cycles for each). 16 s *rRNA* was used as the internal reference gene. The absence of primer dimers was confirmed by the melting curve as well as running the reaction on a 1% agarose gel. Real-Time qRT-PCR results were analyzed via the 2(-Delta Delta C(T)) method^53^.

### Mitobiogenesis assay

To measure the effect of PNA on the mitochondrial biogenesis, In-Cell ELISA Kit (MitoSciences Inc., Eugene, OR, USA) was used as per the manufacturer’s instructions^54^,^55^. Briefly, J774A.1 cells were seeded (approximately 40,000 cells per well) in 96-well plates and allowed to adhere. After overnight incubation, PTAT was added to a final concentrations of 0, 0.1, 1, 10 and 20 μM in duplicate. Ampicillin and linezolid were used at similar concentrations as negative and positive controls, respectively. Cells were allowed to grow for approximately 3 days. Media were removed and cells were washed with PBS, then fixed with 4% paraformaldehyde. After fixing, cells were washed with PBS and permeabilization and blocking processes were done according to the manufacturer’s instructions. Primary antibodies to detect the levels of two proteins (subunit I of Complex IV (COX-I), that is mitochondrial DNA (mtDNA)-encoded), and the 70 kDa subunit of Complex II (SDH-A), a nuclear DNA (nDNA)-encoded were added and incubated for overnight at 4 °C. After incubation, cells were washed with PBS and secondary antibodies were added and incubated at room temperature for one hour. After washing, the expression of SDH-A and COX-1 were measured at 405 nm and 600 nm wavelength, respectively. The ratio between COX-I and SDH-A was calculated and the percent of inhibition of mitochondrial biogenesis was determined.

### Cell culture infection assay

To assess the ability of the PNAs to clear an intracellular infection caused by *L. monocytogenes*, a modified version of a previously described cell culture infection assay was performed^5^. Briefly, J774A.1 cells in 96-well plates were infected with *L. monocytogenes* F4244 at a 1:10 multiplicity of infection (MOI) for 30 minutes and treated with gentamicin to kill extracellular *Listeria*. PNAs were added to a final concentration of 2, 4 and 8 μM and the cells were incubated for 4 hours at 37 °C with 5% CO_2_. After incubation the cells were washed three times with PBS, lysed using 0.1% Triton X-100 and the intracellular bacteria were counted by serial dilution and plating on TSA plates.

### Efficacy of PNA treatment in an animal model of infection

To test the efficacy of PNA *in vivo* we used *Listeria*-infected *C. elegans*. The temperature-sensitive sterile mutant strain *C. elegans* AU37 [sek-1(km4); glp-4(bn2) I] was used for this study. This strain is sterile at room temperature and can lay eggs only at 15 °C. Furthermore, mutation in the sek-1 gene of the p38 mitogen-activated protein kinase pathway (p38 MAPK pathway), makes this particular *C. elegans* strain more susceptible to *Listeria* infection^49^,^56^. Initially, the pathogenicity of three strains of *Listeria* (F4244, J0161 and ATCC19111) was tested in *C. elegans* as described before^24^ to determine the most virulent strain to use for this study. Strain J0161 (most virulent strain) was chosen for testing PNA treatment efficacy *in vivo*. Briefly, adult worms were grown for 5 days, at 15 °C (temperature that permits worms to lay eggs) on NGM agar plates seeded with a lawn of *E. coli* OP50. The eggs were harvested by bleaching^57^ and maintained for 24 hours at room temperature with gentle agitation for hatching. Hatched larvae were transferred to a new NGM plate seeded with *E. coli* OP50 and were kept at room temperature for five days until worms reached the adult stage of growth. Adult worms were collected and washed three times with M9 media in a 1:10 ratio to remove *E. coli* before transfer to LB agar plates seeded with a lawn of *L. monocytogenes* J0161 for infection. After eight hours of infection, worms were collected and washed with M9 buffer five times before incubation with PNAs. Worms were transferred to low-binding microcentrifuge tubes (10 worms per tube). PNAs were added to the tubes in triplicates to achieve a final concentration of either 16 or 32 μM. Sterile water and gentamicin (16 and 32 μM) were used as negative and positive controls, respectively. After treatment for 18 hours, worms were washed five times with M9 buffer. Worms were examined microscopically before lysis for morphological changes and viability (live worms are sinusoidal with movement, whereas dead worms are rigid rods).The worms were lysed by addition of 200 mg of 1.0-mm silicon carbide particles (Biospec Products, Bartlesville, OK) to each tube and vortexing for one minute (the silicon carbide particles disrupt the worms but do not affect bacterial survival). Samples were serially diluted and plated onto TSA plates containing 5 μg/ml nalidixic acid to select for *Listeria*.

### Statistical analysis

Statistical analysis was performed utilizing GraphPad Prism 6.0 (GraphPad Software, La Jolla, CA). Statistical significance was assessed using the two-tailed Student’s *t*-test and ANOVA. *P* values of <0.05 were considered significant. Data are presented as mean ± SD.

## Additional Information

**How to cite this article**: Abushahba, M. F. N. *et al.* Impact of different cell penetrating peptides on the efficacy of antisense therapeutics for targeting intracellular pathogens. *Sci. Rep.* **6**, 20832; doi: 10.1038/srep20832 (2016).

## Supplementary Material

Supplementary Information

## Figures and Tables

**Table 1:** Cell penetrating peptides and antisense constructs used in this study.

Name	Sequence^a^
KFF	KFFKFFKFFK
ANT	RQIKIWFQNRRMKWKK
TAT	GRKKKRRQRRRYK
(RXR)4XB	RXRRXRRXRRXRXB
(RFR)4XB	RFRRFRRFRRFRXB
PKFF	KFFKFFKFFK-O-cgatcattcaaa-NH_2_
PANT	RQIKIWFQNRRMKWKK-O-cgatcattcaaa-NH_2_
PTAT	GRKKKRRQRRRYK-O-cgatcattcaaa-NH_2_
PRXR	RXRRXRRXRRXRXB-O-cgatcattcaaa-NH_2_
PRFR	RFRRFRRFRRFRXB-O-cgatcattcaaa-NH_2_
Free PNA	cgatcattcaaa

^a^Lowercase letters represent the specific 12-nucleotide sequence of the PNA construct; -O- represents linker.

**Table 2:** MICs and MIC_50_ of CPPs and PNAs against clinical isolates of *L. monocytogenes.*

Listeria clinical isolates	MIC^a^ (μM)						MIC_50_^b^(μM)
	F4244	J0161	ATCC 13932	ATCC 19112	ATCC 19111	ATCC 19114	
CPP							
KFF	128	128	128	64	32	64	64
ANT	>128	64	>128	64	32	64	64
TAT	>128	>128	>128	>128	>128	>128	>128
(RXR)4XB	>128	>128	>128	>128	>128	>128	>128
(RFR)4XB	>128	>128	>128	>128	32	128	>128
PNA							
PKFF	32	32	32	32	32	2	32
PANT	16	32	32	32	32	1	32
PTAT	2	4	4	2	4	0.5	2
PRXR	1	4	2	1	1	0.25	1
PRFR	2	16	4	4	8	1	4
Free PNA	>32	>32	>32	>32	>32	>32	>32
Control PNA	>32	>32	>32	>32	32	>32	>32

^a^MIC: The minimum inhibitory concentration of PNA where no bacterial growth was observed.

^b^MIC50: The minimum inhibitory concentration of PNA required to inhibit 50% of *L. monocytogenes* isolates.

**Table 3:** Effect of PNAs on eradicating *L. monocytogenes* F4244 inside infected J774A.1 cells.

Concentrations						
Treatment	2 μM		4 μM		8 μM	
	Log CFU/ml	Log CFU reduction	Log CFU/ml	Log CFU reduction	Log CFU/ml	Log CFU reduction
PKFF	6.77 ± 0.03	−0.031	6.76 ± 0.08	−0.029	6.12 ± 0.32	0.6*
PANT	6.82 ± 0.02	−0.09	6.67 ± 0.05	0.06	4.65 ± 0.34	2.07*
PTAT	5.95 ± 0.02	0.78*	5.19 ± 0.33	1.54*	4.22 ± 0.11	2.51*
PRXR	4.95 ± 0.07	1.78*	3.079	3.65*	cleared	6.73*
PRFR	5.89 ± 0.65	0.84*	5.10 ± 0.28	1.63*	4.29 ± 0.04	2.43*
Control	6.73 ± 0.13	0				

Asterisks indicate values found to be significantly different from control by statistical analysis.
