# Supplementary material for: Impact of different cell penetrating peptides on the efficacy of antisense therapeutics for targeting intracellular pathogens
Source: Sci Rep. 2016 Feb 10;6:20832. doi: 10.1038/srep20832 (PMC4748415; doi:10.1038/srep20832)
Supplement: Supplementary Information [file srep20832-s1.pdf]

Impact of different cell penetrating peptides on the efficacy of antisense therapeutics for targeting intracellular pathogens

Mostafa F.N. Abushahba[1,2], Haroon Mohammad [1], Shankar Thangamani[1], Asmaa A. A. Hussein[2] and Mohamed N. Seleem[1*]

[1]Department of Comparative Pathobiology, College of Veterinary Medicine.

Purdue University, West Lafayette, Indiana, USA, 47907.

[2]Department of Animal Hygiene and Zoonoses, Faculty of Veterinary Medicine,

Assiut University, Assiut, Egypt.

*****Corresponding author: Mohamed N. Seleem. Email: mseleem@purdue.edu

Table S1: Bacterial isolates used for this study:

Bacterial isolate	Description
L. monocytogenes F4244	Invasive strain F4244 CDC. Clinical isolate from patient's cerebrospinal fluid (CSF)
L. monocytogenes J0161	Isolated from a case of human listeriosis linked to a multi-state outbreak from a turkey processing plant, USA. (2000)
L. monocytogenes ATCC 13932	Clinical isolate from cerebrospinal fluid of child with meningitis, Germany
L. monocytogenes ATCC 19112	Clinical isolate from patient's cerebrospinal fluid, Scotland (1935)
L. monocytogenes ATCC 19111	Isolated from poultry, England (1937)
L. monocytogenes ATCC 19114	Isolated from bovine brain, USA (1931)
Methicillin-resistant Staphylococcus aureus (MRSA) USA300	Isolated from a wound in Mississippi, USA. Resistant to erythromycin, methicillin and tetracycline
Escherichia coli OP50	Uracil auxotroph strain with limited growth on nematode growth medium

Table S2: The targeted sequence of the *rpo*A gene for six clinical strains of *L. monocytogenes*.

**Strain**	**Sequence (5′-3′)**
L. monocytogenes F4244	AAGGAGGGTAAA**TTTGAATGATCG**AAATTGAAAAGC
L. monocytogenes J0161	AAGGAGGGTAAA**TTTGAATGATCG**AAATTGAAAAGC
L. monocytogenes ATCC 13932	AAGGAGGGTAAA**TTTGAATGATCG**AAATTGAAAAGC
L. monocytogenes ATCC 19112	AAGGAGGGTAAA**TTTGAATGATCG**AAATTGAAAAGC
L. monocytogenes ATCC 19111	AAGGAGGGTAAA**TTTGAATGATCG**AAATTGAAAAGC
L. monocytogenes ATCC 19114	AAGGAGGGTAAA**TTTGAATGATCG**AAATTGAAAAGC

Table S3. Sequence alignment of *rpoA* 5' terminal region among different *Listeria* species

Organism	Source	Locus tag	Identity [a]	Sequence (5'-3')[b]
Listeria monocytogenes				
J0161	Human CSF	LMOG_01906	100%	GAGGGTAAA**TTTGAATGATCG**AAATTGAAA
EGD-*e*	Rabbit, England	lmo2606	99%	GAGGGTAAA**TTTGAATGATCG**AAATTGAAA
10403S	Human skin lesion, USA	LMRG_02150	99%	GAGGGTAAA**TTTGAATGATCG**AAATTGAAA
07PF0776	Cardiac abscess, human, USA	MUO_13015	95%	GAGGGTAAA**TTTGAATGATCG**AAATTGAAA
F2365	Cheese, USA	LMOf2365_2579	95%	GAGGGTAAA**TTTGAATGATCG**AAATTGAAA
HCC23	Channel catfish	LMHCC_2928	94%	GAGGGTAAA**TTTGAATGATCG**AAATTGAAA
M7	Cow's milk, China	LMM7_2718	94%	GAGGGTAAA**TTTGAATGATCG**AAATTGAAA
Listeria innocua				
Clip11262	Food, Morocco	lin2755	87%	GAGGGTAAA**TTTGAATGATCG**AAATTGAAA
Listeria ivanovii				
PAM 55	Sheep, Spain	LIV_2517	84%	GAGGGTAAA**TTTGAATGATCG**AAATTGAAA
Listeria welshimeri				
SLCC5334	Decaying vegetation, USA	lwe2556	86%	GAGGGTAAA**TTTGAATGATCG**AAATTGAAA
Listeria seeligeri				
SLCC3954	Soil, Germany	lse_2511	84%	GAGGGTAAA**TTTGAATGATCG**AAATTGAAA

[a] The identities between different *Listeria* strains were determined by using BLAST (Basic Local Alignment Search Tool).

[b] *rpo*A gene 5' terminal region among *Listeria* species showing the targeted area of interest in boldface type and the start codon ATG is bold and underlined.

Table S4: Effect of PNAs on pure culture of L. monocytogenes F4244

PNA	PNA Concentrations											
	1 μM		2 μM		4 μM		8 μM		16 μM		32 μM	
	Log CFU/ml	Log CFU reduction	Log CFU/ml	Log CFU reduction	Log CFU/ml	Log CFU reduction	Log CFU/ml	Log CFU reduction	Log CFU/ml	Log CFU reduction	Log CFU/ml	Log CFU reduction
**PKFF**	9.40±0.08	-0.009	9.35±0.05	0.041	9.26±0.07	0.129	8.93±0.35	0.46	8.40±0.16	0.98*	6.63±0.59	2.78*
**PANT**	9.30±0.06	0.095	9.27±0.21	0.119	9.14±0.05	0.25	8.09±0.95	1.30*	6.73±0.48	2.66*	Cleared	9.39*
**PTAT**	8.28±0.37	1.1*	5.94±0.42	3.45*	Cleared	9.39*	Cleared	9.39*	Cleared	9.39*	Cleared	9.39*
**PRXR**	6.87±0.84	2.51*	3.15±0.21	6.24*	Cleared	9.39*	Cleared	9.39*	Cleared	9.39*	Cleared	9.39*
**PRFR**	9.26±0.09	0.13	7.48±0.69	1.91*	6.10±0.63	3.29*	Cleared	9.39*	Cleared	9.39*	Cleared	9.39*
**Control**	9.39±0.04	0										

Asterisks indicate values found to be significantly different from water by statistical analysis.

Table S5: Selected genes used for this study and their proposed function

Gene	Locus tag	Proposed function
*rpo*A	lmo2606	DNA-dependent RNA polymerase catalyzes the transcription of DNA into RNA using the four ribonucleoside triphosphates as substrates [1]
hly	lmo0202	Encodes Listeriolysin O (LLO), a sulfhydryl-activated toxin that causes cytolysis by forming pores in cholesterol-containing host membranes [2]
*plc*A	lmo0201	Encodes phosphatidylinositol-specific phospholipase C (PI-PLC) which cleaves glycosylphosphatidylinositol (GPI) and phosphatidylinositol (PI) anchors but not PI phosphates. Important factor in Listeria pathogenesis that works in conjunction with LLO lyse vacuoles inside host cells [3-6]
*plc*B	lmo0205	Encodes phosphatidylcholine-phospholipase C (PC-PLC) which plays an important role in the infection process. It assists LLO in efficient lysis of the two-membrane vacuoles that surround the bacteria after direct cell-to-cell spread [3-5, 7]
16s rRNA	M645_14975	Housekeeping gene [34, 35]

Table S6: Effect of PNAs on growth of *L. monocytogenes* J0161 in infected *C. elegans*.

Concentrations				
**Treatment**	**16 μM**		**32 μM**	
	Log CFUs/worm	Log CFUs reduction	Log CFUs/worm	Log CFUs reduction
**PKFF**	3.43±0.2	0.71*	3.35±0.25	1.03*
**PANT**	3.88±0.15	0.27	2.49±0.48	1.89*
**PTAT**	3.04±0.36	1.11*	1.7±0.6	2.67*
**PRXR**	2.24±0.99	1.91*	0	4.38*
**PRFR**	2.85±0.60	1.3*	1.86±0.26	2.52*
**Free PNA**	4.29 ±0.17	-0.13	4.39±0.18	-0.012
**Gentamicin**	3.52±0.55	0.63*	2.18±0.6	2.2*
**Water**	4.15±0.06	0	4.38±0.12	0

Asterisks indicate values found to be significantly different from water by statistical analysis.

Table S7: Primers used in this study

Primer name	Target gene	Sequence (5′-3′)	Amplicon size (bp)
RpoA-seqF	*rpo*A	ACAAGCAGCTGGTCTTGAAGTAAC	386
RpoA-seqR	*rpo*A	ACCTTCAATTACAGAAAACTCATG	
RpoA-F	*rpo*A	GTCCTGGTGTAGTAACTGCAG	145
RpoA-R	*rpo*A	GCAGGTGTGTAACCACGACC	
Hly-F	*hly*	CATGGCACCACCAGCATCTC	136
Hly-R	*hly*	CACTGCATCTCCGTGGTATAC	
PlcA-F	*plc*A	CAAGATGACTACAATGGTCCG	149
PlcA-R	*plc*A	GCTGCAGCATACTGACGAGG	
PlcB-F	*plc*B	ATCGGTGACTGATTACCGAG	147
PlcB-R	*plc*B	TATGCACAGTGGTAGCCTGG	
HK-F	*16s rRNA*	GCCTACCAAGGCAACGATGC	145
HK-R	*16s rRNA*	CATACACGCGGCGTTGCTC	
